# Effects of PERMISSive lung-protective ventilation on outcome in critically ill, invasively ventilated patients (PERMISS): protocol for a feasibility and safety randomised clinical trial

**DOI:** 10.1136/bmjresp-2026-004532

**Published:** 2026-09-17

**Authors:** Alessandro Caroli, Amarens Hoogenboom, Guillermo Bugedo, Felipe L Damiani, Juliana Ferreira, Marcelo Gama de Abreu, Jan-Willem Geerts, Bart P Grady, Stephanie S List, David M P van Meenen, Luis Morales-Quinteros, Frederique Paulus, Ary Serpa Neto, Marcus J Schultz, Arthur S Slutsky, Kanwalpreet Sodhi, Louise Urlings-Strop, Laura A Buiteman-Kruizinga, PERMISS collaborative group, PROVE network

**Affiliations:** 1 Intensive Care, Amsterdam UMC Locatie AMC, Amsterdam, The Netherlands; 2 Department of Intensive Care, Austin Hospital, Heidelberg, Victoria, Australia; 3 Department of Physical Care, Pontificia Universidad Católica de Chile, Santiago, Chile; 4 Department of Physical Therapy, Pontificia Universidad Católica de Chile, Santiago, Chile; 5 Respiratory ICU, University of Sao Paulo, São Paulo, Brazil; 6 Department of Cardiothoracic Anesthesia, Anesthesiology Institute, Cleveland Clinic, Cleveland, Ohio, USA; 7 Department of Intensive Care, Reinier de Graaf Hospital, Delft, The Netherlands; 8 Department of Intensive Care, Hospital Group Twente, Almelo, The Netherlands; 9 Department of Intensive Care, Dijklander Hospital, Hoorn, The Netherlands; 10 Department of Anesthesiology, Amsterdam University Medical Centres, Amsterdam, The Netherlands; 11 Intensive Care Department, Vall d’Hebron University Hospital, Barcelona, Spain; 12 Department of Critical Care, Austin Hospital, Heidelberg, Victoria, Australia; 13 Monash University Australian and New Zealand Intensive Care Research Centre, Melbourne, Victoria, Australia; 14 Department of Anaesthesia, Medical University of Vienna, Vienna, Austria; 15 Department of Anaesthesiology, Rescue- and Pain Medicine, Cantonal Hospital St. Gallen, St Gallen, Switzerland; 16 Interdepartmental Division of Critical Care, University of Toronto, Toronto, Ontario, Canada; 17 Department of Critical Care, Deep Hospital, Ludhiana, India; 18 Intensive care, Reinier de Graaf Gasthuis, Delft, The Netherlands

**Keywords:** ARDS, Assisted Ventilation, Critical Care

## Abstract

**Introduction:**

Mechanical power (MP) of ventilation is a summary variable of determinants of ventilator-induced lung injury and is consistently associated with worse outcomes. Lung-protective ventilation (LPV) using a lower respiratory rate (RR) is an appealing strategy to reduce MP, but it is unclear whether reducing RR is feasible and safe in critically ill patients under invasive mechanical ventilation. Therefore, we test the hypothesis that a ventilation strategy that targets the lowest acceptable RR is feasible and safe.

**Methods and analysis:**

The ‘Effects of PERMISSive Lung-protective Ventilation on Outcome in Critically Ill, Invasively Ventilated Patients (PERMISS)’ pilot study is an international, multicentre randomised clinical feasibility and safety trial in critically ill, invasively ventilated adult patients with an expected duration of ventilation >24 hours. Patients will be randomly assigned to ‘permissive’ LPV with a lower RR, within safety limits of arterial partial pressure of carbon dioxide (PaCO_2_) and pH, compared with conventional LPV with RR to target normal PaCO_2_ and pH. The primary endpoint is the difference in RR between the two groups from the start of ventilation until the first extubation. Secondary endpoints include safety, assessed by the incidence of unacceptable hypercapnia (PaCO_2_>70 mm Hg and pH<7.20), severe hypoxaemia (arterial partial pressure of oxygen (PaO_2_)<60 mm Hg), ventilator-associated complications and compliance with the protocol and quality of data collection.

**Ethics and dissemination:**

The current version of the protocol is V.3 (issued on 11 December 2025). The trial was approved by the medical research ethics committee of Leiden Den Haag Delft (approval number NL-009624.P25.028) on 2 June 2025, including all amendments, and was registered on www.ClinicalTrials.gov (NCT07077174). The PERMISS pilot is currently recruiting patients.

**Trial registration number:**

NCT07077174.

**WHAT IS ALREADY KNOWN ON THIS TOPIC**

- Retrospective and observational studies reported that respiratory rate (RR) is an important contributor to the intensity of ventilation, expressed as mechanical power (MP) of ventilation. Mechanical ventilation with high MP is associated with worse outcomes. A ventilation strategy with lower RR, accepting a higher arterial partial pressure of carbon dioxide (PaCO_2_) and lower arterial pH (pHa), referred to as ‘permissive’ lung-protective ventilation (LPV), results in reduced intensity of ventilation.

**WHAT THIS STUDY ADDS**

- The ‘Effects of PERMISSive Lung-protective Ventilation on Outcome in Critically Ill, Invasively Ventilated Patients’ pilot study evaluates whether a permissive LPV strategy using a lower RR is feasible and safe compared with conventional LPV using a higher RR. The main hypothesis is that a ventilation strategy targeting a low RR, according to the highest acceptable PaCO_2_ but limited to the lowest acceptable pH, will reduce both the RR and the intensity of ventilation without increasing the incidence of unacceptable episodes of extreme hypercapnia, severe hypoxaemia or ventilation-associated complications compared with the current standard of care.

**HOW THIS STUDY MIGHT AFFECT RESEARCH, PRACTICE OR POLICY**

- The results will directly inform the design of the future large-scale randomised clinical trial to investigate whether ventilation with lower RR might be superior to conventional ventilation in terms of clinical outcomes.

## Introduction

 Since the landmark ‘Ventilation with lower tidal volumes as compared with traditional tidal volumes for acute lung injury and acute respiratory distress syndrome’ trial, low tidal volume (V_T_) has become the cornerstone of lung-protective ventilation (LPV), evolving into its broad and indiscriminate application.^1^ Thereafter, the pathophysiology of ventilator-induced lung injury (VILI) has been recognised as a complex phenomenon governed by multiple mechanistic variables whose interactions remain incompletely understood.^2^ Importantly, focusing on individual ventilatory parameters may obscure relevant trade-offs. For example, reductions in V_T_ are often accompanied by compensatory increases in respiratory rate (RR) to maintain minute ventilation, which may offset potential benefits.^3 4^ In this context, the mechanical power (MP) of ventilation has been proposed as a summary variable of the determinants of VILI.^5^ Accumulating evidence demonstrates the association between elevated MP and worse clinical outcomes: ventilation with higher MP is related to increased mortality, prolonged ventilation duration and prolonged intensive care unit (ICU) stay in critically ill patients.^6–10^

RR is an important contributor to MP.^11 12^ Both animal experiments and human epidemiological studies have demonstrated the substantial role of RR as a potential modifiable factor contributing to VILI.^13–15^ A ventilation strategy with a lower RR, accepting higher arterial partial pressure of carbon dioxide (PaCO_2_) and lower arterial pH (pHa), referred to as ‘permissive lung-protective ventilation’, results in reduced intensity of ventilation and may potentially improve outcomes. In a recent post hoc analysis using individual patient data from 1732 critically ill patients receiving standard LPV from three randomised clinical trials (RCTs), variations in MP were more strongly correlated with changes in RR than with changes in V_T_ or airway pressures.^4^ Moreover, lower MP and lower RR were associated with reduced mortality. These observational findings, along with the lack of robust prospective data, render RR a promising yet unexplored variable for investigation in an RCT.

This article presents the protocol for the ‘Effects of PERMISSive Lung-protective Ventilation on Outcome in Critically Ill, Invasively Ventilated Patients (PERMISS)’ pilot trial. The proposed study aims to assess the feasibility and safety of a ‘permissive’ LPV using low RRs and compare it to conventional LPV in adult critically ill patients. This study will inform the design of a future RCT that will evaluate the efficacy of lower RR in improving patient-centred outcomes. We hypothesise that permissive LPV is safe and feasible in this group of patients.

## Methods and analysis

### Trial objective, design and setting

**Figure 1:**
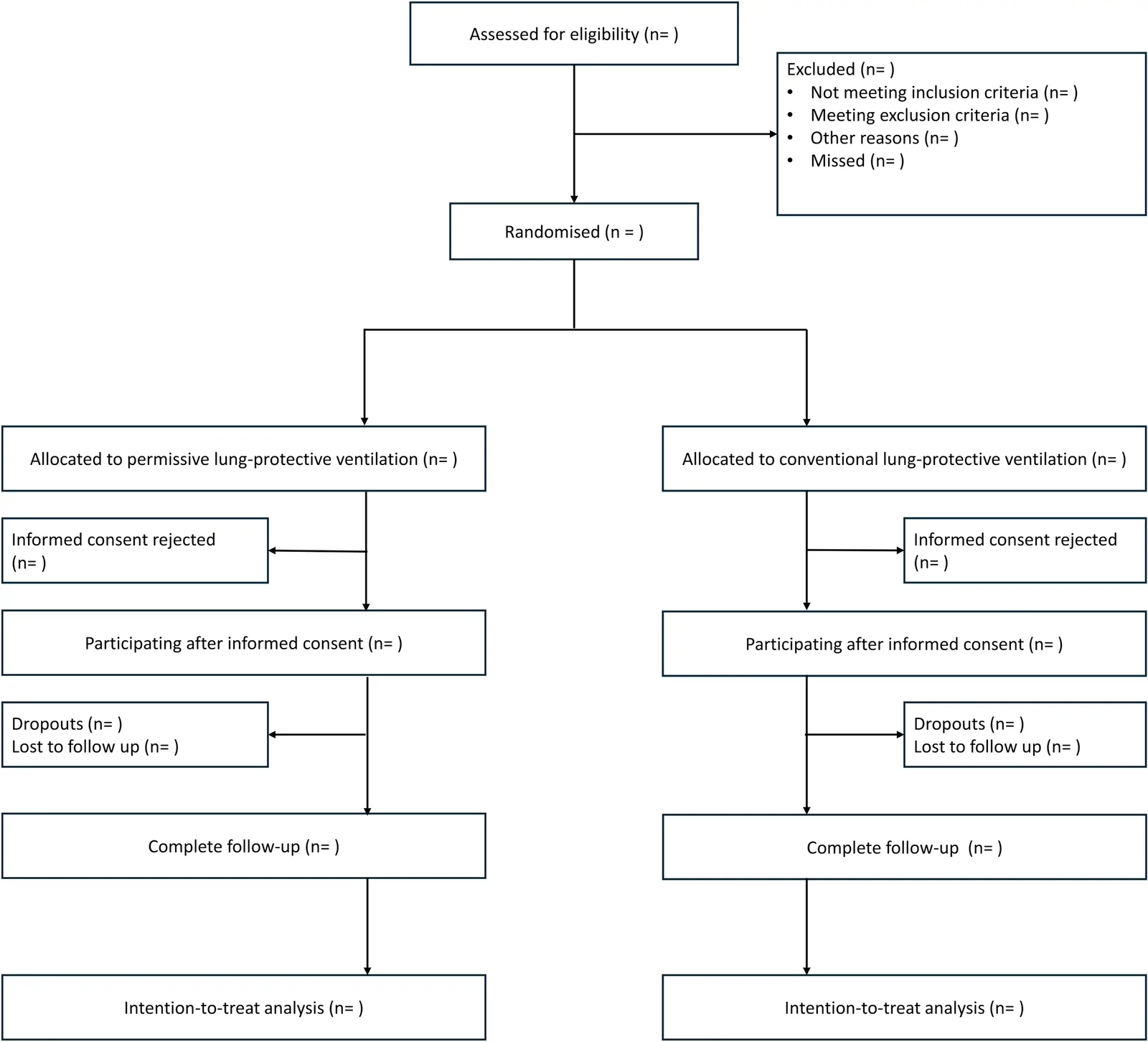
Proposed flow diagram of participants.

The ‘PERMISS’ pilot study is an international, investigator-initiated, multicentre, prospective, two-group, randomised clinical feasibility and safety trial in critically ill, invasively ventilated adult ICU patients with an anticipated duration of invasive mechanical ventilation (IMV) of >24 hours. PERMISS pilot’s primary aim is to test whether a ‘permissive’ LPV strategy can effectively reduce RR and MP compared with a conventional LPV strategy. Secondary aims include assessment of protocol compliance, the safety of the intervention and feasibility of data collection. Patients are enrolled across four centres in the Netherlands (Reinier de Graaf Hospital, Delft, the Netherlands; Hospital Group Twente, Almelo, the Netherlands; Dijklander Hospital, Hoorn, the Netherlands and Amsterdam University Medical Centre, Amsterdam, the Netherlands) and one in Spain (Vall d’Hebron, Barcelona, Spain) and randomised to one of the two ventilation strategies (figure 1).

### Study population

The PERMISS pilot enrols intubated and ventilated ICU adult patients with an expected duration of invasive ventilation >24 hours, based on clinical judgement and following previous similar trials.^16–18^ Patients are screened for eligibility and randomised within 1 hour after the initiation of IMV, or within 1 hour after ICU admission if IMV starts before entering the ICU. Patients receiving IMV>1 hour in the ICU or receiving IMV>6 hours directly preceding the current ICU admission are excluded. Patients with contraindications to hypercapnia, acute cardiac ischaemia, suspected or confirmed increased intracranial pressure or who are diagnosed with moderate to severe chronic obstructive pulmonary disease (Global Initiative for Chronic Obstructive Lung Disease (GOLD) class III or IV) are excluded. Other exclusion criteria are age<18 years, confirmed or suspected pregnancy, receiving or planned to receive extracorporeal membrane oxygenation, admission for terminal care, anamnesis of any neurological diagnosis that can prolong IMV, participation in trials with similar endpoints, previous randomisation in this study and absence of written deferred informed consent.

### Sample size calculation

Based on previous literature,^19 20^ we assumed an expected mean difference of 7.5 breaths per min, with an SD (σ) of 10. Accepting a probability of type II error of 10% (power=90%) and a probability of type I error of 5% (α=0.05), a minimum of 38 participants per group are required to detect a significant mean reduction of RR with a two-sample t-test. Accounting for an expected dropout rate of 10%, we will enrol a total of 84 patients (42 per arm). As a pilot study, recruitment may be stopped early if additional inclusions are unlikely to yield new relevant information on feasibility, data completeness or key parameter variability, as determined by the steering committee.

### Deferred consent

In this study, a deferred informed consent procedure is used, and we appeal to the emergency procedure for consent in medical research as stated in Article 6, paragraph 4 of the WMO. Nevertheless, written informed consent from the legal representative is obtained as soon as possible and always within 72 hours after randomisation. If consent is not obtained within this period or is declined, the patient is excluded, and all data are discarded. A second informed consent is sought from the patient in retrospect after recovery.

### Intervention and comparison

**Figure 2:**
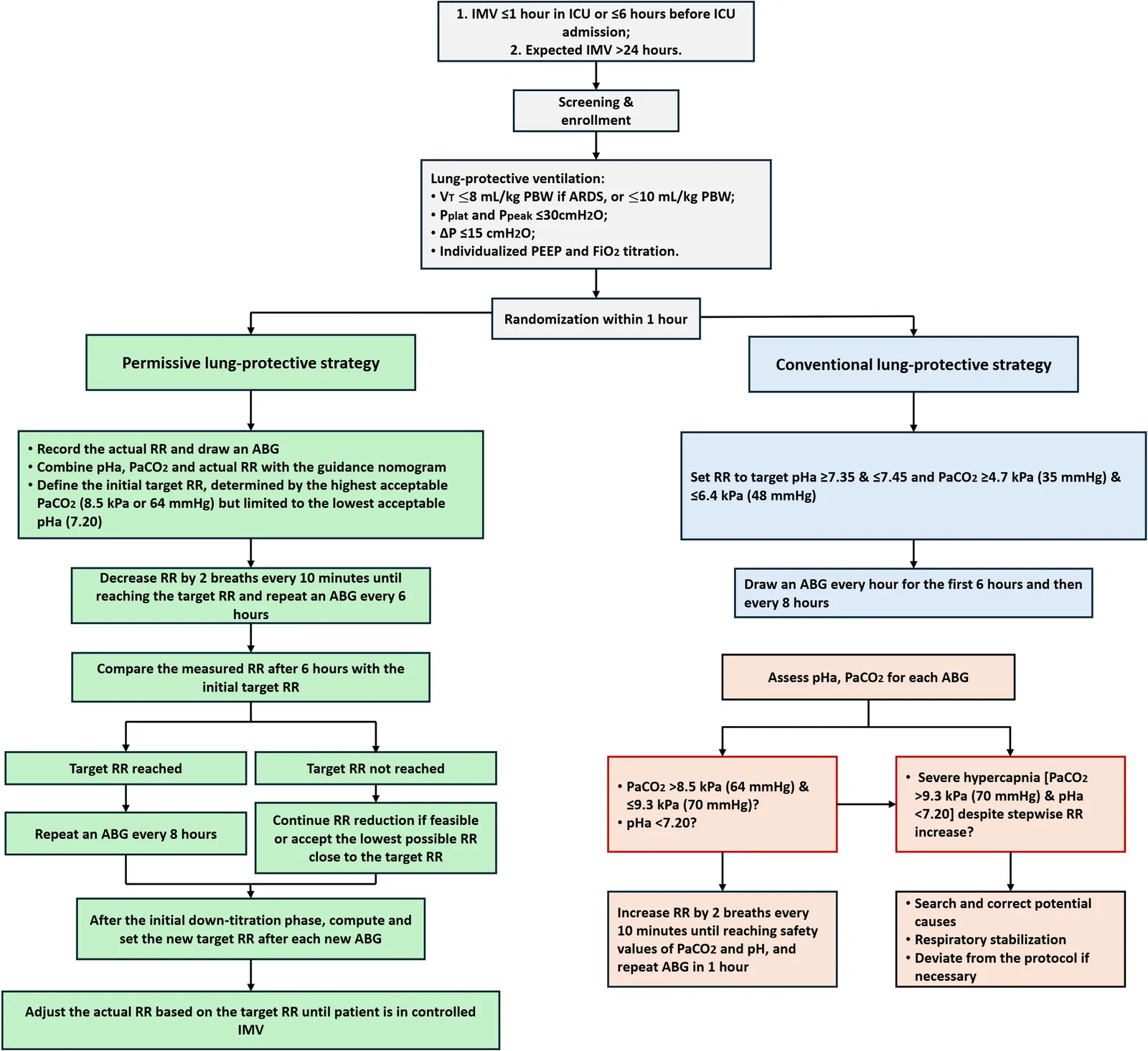
Flowchart of the two ventilation strategies under comparison. ABG, arterial blood gas; ARDS, acute respiratory distress syndrome; cmH₂O, centimeters of water; FiO₂, fraction of inspired oxygen; ICU, intensive care unit; IMV, invasive mechanical ventilation; PaCO₂, arterial partial pressure of carbon dioxide; PBW, predicted body weight; PEEP, positive end-expiratory pressure; P_peak_, peak pressure; P_plat_, plateau pressure; pHa, arterial pH; RR, respiratory rate; V_T_, tidal volume; ΔP, driving pressure.

**Figure 3:**
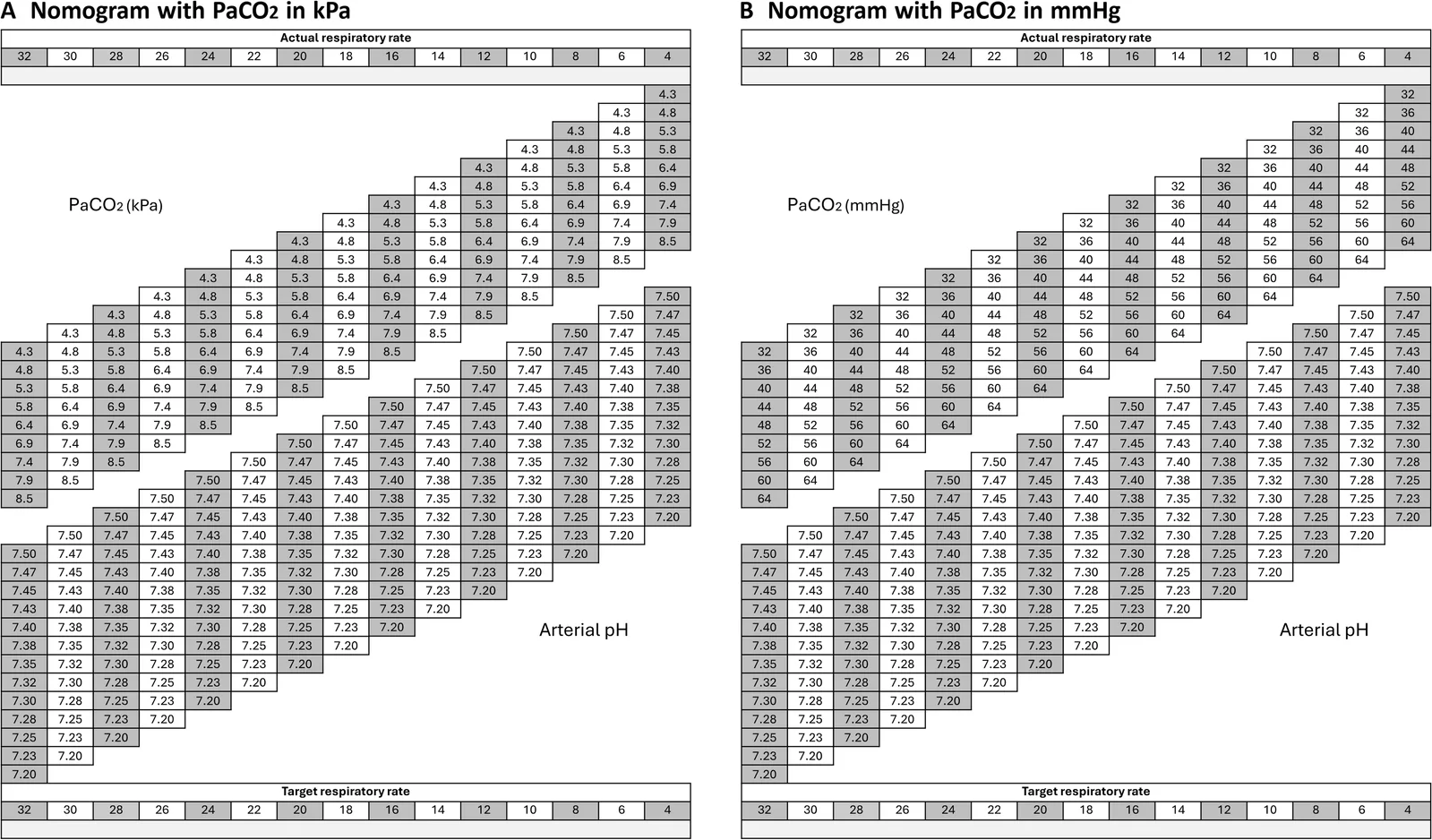
Nomogram for assisting RR titration in the permissive LPV group. The nomogram provides the estimated RR within the highest acceptable PaCO_2_ of 8.5 kPa (64 mm Hg) and the lowest acceptable pHa of 7.20 according to the actual RR, PaCO_2_ and pHa. The target RR is determined by combining the ABG results and the RR measured at the time of sampling with this guidance nomogram. First, the measured PaCO_2_ and pHa are located in the RR column corresponding to the actual RR. Second, the RR corresponding to PaCO_2_ equal to 8.5 kPa (64 mm Hg) is identified by moving across the row of the measured PaCO_2_, and the RR corresponding to pHa equal to 7.20 is identified with the same approach. Finally, the target RR is the highest RR between the one corresponding to PaCO_2_ equal to 8.5 kPa (64 mm Hg) and the one corresponding to pHa equal to 7.20, or the minimum possible value of 4 breaths per minute. (A) shows the nomogram to be used for centres measuring PaCO_2_ in kPa, and (B) shows the nomogram to be used for centres measuring PaCO_2_ in mm Hg. ABG, arterial blood gas; LPV, lung-protective ventilation; PaCO_2_, arterial partial pressure of carbon dioxide; pHa, arterial pH; RR, respiratory rate.

Patients included in this study will be randomised to a permissive LPV strategy or a conventional LPV strategy (figure 2). The goal in the permissive LPV group is to achieve the lowest possible RR according to predefined safety limits; that is, the highest acceptable PaCO_2_ of 64 mm Hg (8.5 kPa), but limited by the lowest acceptable pHa of 7.20. The lowest possible RR is determined by combining the arterial blood gas (ABG) results and the RR measured at the time of sampling with a guidance nomogram (figure 3). The latter provides the estimated RR within the highest acceptable PaCO_2_ and the lowest acceptable pHa thresholds according to the actual RR, PaCO_2_ and pHa and was derived from previous studies.^19 20^ During controlled ventilation, the RR is decreased in steps of 2 breaths every 10 min while monitoring end-tidal CO_2_ partial pressure (etCO_2_), and ABGs are repeated hourly for at least 6 hours or until the target RR is reached. If etCO_2_ remains stable, an ABG may be omitted, provided the reason is documented. Thereafter, ABGs are repeated at least every 8 hours, and a new target RR is re-evaluated according to the guidance. The minimum RR that can be set is 4 breaths per minute.

If the pHa falls below 7.20 or there are other reasons why it is necessary to increase the RR, as judged by the attending physician, the RR is gradually increased in steps of 2 breaths every 10 min and monitored with hourly ABGs. The RR can be increased more quickly if the attending physician thinks it is in the patient’s best interest. If moderate alkalosis (pHa>7.50) or severe acidosis (pHa<7.20) is present at randomisation, pHa must be decreased to ≤7.50 or increased to ≥7.20 based on local protocols before the study intervention can be delivered. This approach is continued until the patient is transitioned to spontaneous breathing. Once the patient is breathing spontaneously, attempts to reduce spontaneous RR are made by increasing the pressure support by 1 cmH_2_O every 30 min, always keeping V_T_, peak pressure (P_peak_) and driving pressure (ΔP) within predefined lung-protective ranges (described in the next paragraph).

In patients randomised to conventional LPV, the RR is set according to standard of care to target a normal PaCO_2_ (4.7–6.4 kPa or 35–48 mm Hg) and a normal pHa (7.35–7.45), or according to clinical targets if required to maintain LPV. Permissive hypercapnia or lower pHa are accepted if these targets cannot be achieved while maintaining LPV (eg, severe acute respiratory distress syndrome (ARDS)). ABGs are collected hourly for the first 6 hours after randomisation and at least every 8 hours thereafter.

### Standard ventilation management

Standard and commonly used ventilator modes are recommended. Semiautomated or fully automated closed-loop ventilator modes are not allowed at any time. In both randomisation groups, protective ventilation settings must be maintained: V_T_≤8 mL/kg of predicted body weight (PBW) for patients with ARDS or ≤10 mL/kg of PBW for patients without ARDS, and P_peak_≤30 cm H_2_O or plateau pressure ≤30 cm H_2_O. V_T_ must be adjusted to keep the ΔP<15 cm H_2_O. Positive end-expiratory pressure and fraction of inspired oxygen are targeted according to individual patients and local protocols.

### Standard procedures beyond ventilator management

Sedation strategies follow local guidelines, with no recommendations regarding the preferred analgo-sedation strategy or specific indications for administering paralysing agents (neuromuscular blocking agents (NMBAs)). The level of sedation is routinely assessed and reported. Sedation and muscle paralysis should not be used to facilitate permissive ventilation or to better tolerate hypercapnia. Clinicians and nurses are responsible for checking patient readiness for transition to spontaneous breathing and extubation, according to standard clinical practice and local guidelines.

### Study endpoints

The primary feasibility endpoint is the difference in RR between the two groups from the start of ventilation until the first extubation. Secondary endpoints are the safety and feasibility of the study protocol. Safety is assessed by the occurrence of unacceptable hypercapnia (PaCO_2_>70 mm Hg or 9.3 kPa and pH<7.20) and hypoxaemia (PaO_2_<60 mm Hg or 8 kPa) and the incidence of ventilator-associated complications defined as air leaks, severe atelectasis, ventilator-associated pneumonia and acute cor pulmonale. Feasibility is further assessed through compliance with the intervention strategy and data collection.

### Randomisation

Patients will be randomly assigned in a 1:1 ratio to permissive or conventional LPV via a computer-generated allocation sequence with permuted blocks, with a maximum size of 8 and stratified by centre. Randomisation is performed by the local investigator via a password-protected randomisation tool in the Castor Electronic Data Capture (EDC) platform (www.castoredc.com).

### Follow-up and data collection and handling

Table 1 shows the schedule of enrolment, interventions and assessments. Demographic and baseline data, as well as data on disease severity, are collected within the first 24 hours of ICU admission. Data collection includes gender, age, height, weight, reason for ICU admission, reason for IMV, Acute Physiology and Chronic Health Evaluation IV score and Sequential Organ Failure Assessment. Data on ventilator parameters are collected hourly for the first 6 hours after randomisation and, thereafter, three times per day at fixed timepoints during the first 5 days. From day 6, data will be collected once a day at a fixed timepoint. Ventilator parameters are additionally collected via an automated data-capturing system connected to the ventilator before randomisation and for 24 hours. Data on blood gas analyses are collected every hour for the first 6 hours; then, they will be repeated three times per day at fixed timepoints. Additional data include information on the type and dose of sedatives and NMBAs up to day 5, Richmond Agitation-Sedation Scale, body temperature, daily type and amount of nutrition up to day 5 and dose, date and time of sodium bicarbonate administration up to day 5. Exploratory follow-up data regarding duration of ventilation, ICU and hospital discharge and death are collected at day 28 and at day 90 from randomisation.

**Table 1:** Schedule of enrolment, intervention and assessment

	Study period								
	Screening	Randomisation	Postrandomisation					Follow-up	
	Start of ventilation or ICU admission	≤1 hour after starting ventilation	First 6 hours after enrolment	24 hours	72 hours	Day 0–5	Day 5 to extubation/death/day 28	Day 28	Day 90
Enrollment									
Eligibility screen	**X**								
Randomisation		**X**							
Deferred consent*			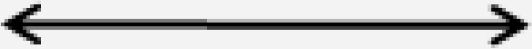						
Intervention									
Permissive lung-protective strategy			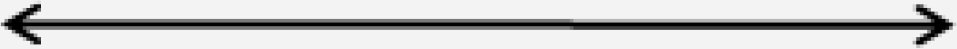						
Conventional lung-protective strategy			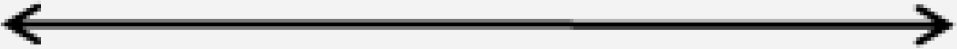						
Assessment									
Demographic, baseline and severity-of-illness characteristics	**X**								
Ventilation data	**X**	**X**	**X (hourly**)	**X**	**X**	**X (three times a day**)	**X (one time a day**)		
Blood gas analysis data	**X**	**X**	**X (hourly**)	**X**	**X**	**X (three times a day**)	**X (three times a day**)		
Continuous automated data		**X**	**X**	**X**					
Sedative, neuromuscular blocking agent and nutrition data		**X**	**X**	**X**	**X**	**X**			
Body temperature		**X**	**X**	**X**	**X**	**X**	**X**		
Sodium bicarbonate administration		**X**	**X**	**X**	**X**	**X**			
AEs†			**X**	**X**	**X**	**X**	**X**		
Ventilator-associated pulmonary complications‡			**X**	**X**	**X**	**X**	**X**		
Intubation and duration of ventilation								**X**	**X**
ICU and hospital discharge								**X**	**X**
Life status and patient location								**X**	**X**

* Deferred consent is obtained as soon as possible but never later than 72 hours after randomisation.

† AEs are defined as unacceptable hypercapnia (PaCO_2_>9.3 kPa or 70 mm Hg) and severe hypoxaemia (PaO_2_<8 kPa or 60 mm Hg), or any other unexpected AE related to the intervention.

‡ Ventilator-associated pulmonary complications include air leaks, severe atelectasis, ventilator-associated pneumonia and acute cor pulmonale.

AE, adverse event; ICU, intensive care unit; PaCO_2_, arterial partial pressure of carbon dioxide; PaO_2_, arterial partial pressure of oxygen.

### Study dropouts and missing data

Participation in the PERMISS pilot study is voluntary. Patients will be classified as dropouts if they were randomised but cannot start the strategies under comparison due to unforeseen circumstances or when deferred consent is not obtained. Dropout patients will be replaced. The patient or patient’s representative can withdraw consent for collecting study data and for participation in the study at any time during the trial and without giving any reasons. No or minimal losses to follow-up for the primary and secondary outcomes are anticipated. If any missing data are found for the primary outcome, a sensitivity analysis using multiple imputations will be carried out.

### Data management

The database in Castor EDC will be locked and stored in a password-protected archive as soon as all data are entered and all attempts to resolve discrepant or missing data are made. Identifying data will be separated from research data and replaced with assigned codes. Direct identifying data will be used only to contact participants and will be accessible only to local investigators. All data will be stored for the length of the study and for 15 years afterwards. Data analysis will be performed with a de-identified dataset and in a blinded fashion. The PERMISS pilot’s results will be used to inform a future RCT and to produce results for scientific journal publication.

### Statistical analysis

A finalised statistical analysis plan will be updated and made available before cleaning and closing the database. All statistical analyses will be conducted on a modified intention-to-treat basis, with patients analysed according to their assigned treatment arms, except for cases lost to follow-up or withdrawal of informed consent. Variables will be expressed as counts and percentages, means and SD or medians and IQRs. Primary and secondary endpoints will be assessed using a two-sided superiority hypothesis test with a significance level of 0.05 and presented with a two-sided 95% CI.

The primary endpoint, as well as other continuous longitudinal variables, will be compared using mixed-effects models and presented as mean or median differences with 95% CIs. Distribution plots comparing these variables between groups will be constructed, presenting the data at each time point. Secondary endpoints of this pilot trial will be assessed to evaluate whether the target RR was reached and maintained during the ventilation period in the permissive LPV group and how often a patient was switched back to the other ventilation group. Counts and percentages of protocol deviations, with their reasons, will also be reported. Missing data will be reviewed and reported to assess the feasibility of data collection. The occurrence of unacceptable hypercapnia and the occurrence of severe hypoxaemia will be reported as rates of events. Incidence of any ventilator-associated complications will be reported. Clinical outcomes analysis will be exploratory, with a follow-up at day 28 and at day 90.

## Ethics and dissemination

### Ethics

The current version of the protocol is V.3 (issued on 11 December 2025), and was registered on www.ClinicalTrials.gov (NCT07077174). The PERMISS pilot is currently recruiting patients.

### Trial organisation

This study is an investigator-initiated trial, sponsored by the Reinier de Graaf Hospital, Delft, the Netherlands. The sponsor, in accordance with Section 10, subsection 4 of the WMO, can halt the study at any time if there are sufficient grounds that continuation of the study will jeopardise subjects’ health or safety. The steering committee consists of the principal investigator, the coordinating investigator, local investigators and international experts in ventilation who contribute to the design and revisions of the study protocol.

### Data safety monitoring board

An independent Data Safety Monitoring Board (DSMB), consisting of four individuals with extensive clinical research experience in the field, oversees the study conduct and the possible side effects of the study treatment. The role of this committee is to ensure that the safety of patients involved in the study is protected. The DSMB has reviewed and approved the study protocol before the start of the trial in the first meeting. The DSMB will review the overall status of the programme, the number of patients enrolled, the adherence to the protocol and the safety of both ventilation strategies in multiple meetings throughout the trial conduction. Any reports and advice from the DSMB will be sent to the sponsor of the study.

### Safety reporting

Reasons for protocol deviation are reported and discussed with the DSMB along with adverse events (AEs) and serious AEs (SAEs). As both ventilation strategies tested in this study are routinely used as standard care in participating ICUs, related SAEs or AEs are not expected. Furthermore, the study population consists of critically ill patients, with a high incidence of death or life-threatening events due to the severity of their illness. Therefore, only unexpected AEs that are related to the intervention and secondary endpoints that incorporate ventilation-specific complications will be reported to the DSMB every 6 months, blinded for treatment groups.

### Patient and public involvement

It was not possible to involve patients or the public in the design, conduct, reporting or dissemination plans of this protocol.

## Discussion

The PERMISS pilot will evaluate whether a permissive LPV strategy, using a lower RR, is feasible and safe compared with conventional LPV, using a higher RR. Additional endpoints include assessing adherence to the study protocol and the quality of data collection. We hypothesise that a ventilation strategy targeting a low RR, according to the highest acceptable PaCO_2_ but limited to the lowest acceptable pHa, combined with a stepwise reduction of the RR and frequent ABG monitoring, will reduce both the RR and the MP. We hypothesise that the strategy will not increase unacceptable episodes of extreme hypercapnia, severe hypoxaemia or ventilation-associated complications. Assessing the safety of sustained application throughout invasive ventilation remains of outstanding importance to inform the large trial, as our hypothesis is based on short-term physiological studies, predefined safety limits, stepwise titration and frequent ABG monitoring used in the present protocol. An additional key objective is to assess the feasibility of implementing the intervention within the first hour of IMV in the ICU, for which we provided specific elements such as the deferred informed consent procedure, the 24/7 availability of the Castor ECD platform and the integration of patients’ screening in the routine ICU workflow with the support of clinical staff. If the permissive strategy proves safe and the protocol demonstrates adequate adherence and data quality, the results will directly inform the design of the future large-scale RCT; if specific protocol elements prove unfeasible, they will be refined based on the findings from this pilot phase.

In the current era of low-V_T_ and low-airway pressure LPV, exploring additional contributors to VILI may help optimise mechanical ventilation. Evidence that high RR might have harmful effects has emerged from preclinical studies in animals and observational studies in humans.^13 15 21–26^ The ’Large observational study to understand the global impact of severe acute respiratory failure’ (LUNG SAFE) identified RR as a potential modifiable independent factor associated with hospital mortality.^14^ Furthermore, RR is a strong determinant of MP, which is associated with patient-centred outcomes.^4–9 15^ A recent study demonstrated that reducing RR over a 12-hour period in patients with COVID-19-related ARDS is both feasible and effective in lowering MP, without causing significant gas exchange derangements or increasing the expression of VILI biomarkers.^19^ These findings suggest that a ‘permissive’ approach to LPV, characterised by lower RR, could represent a promising strategy. However, it remains uncertain whether sustained reductions in RR are both feasible and safe during the total duration of IMV: the PERMISS pilot study is designed to address this question.

A consequence of lowering the RR while maintaining a protective V_T_ is a reduction in alveolar ventilation and a consequent increase in PaCO_2_. Despite that, changes in PaCO_2_ do not correlate linearly with changes in RR due to the influence of V_T_ and dead space, and the longer inspiratory and expiratory phases achieved with lower RR could achieve sufficient gas exchange, limiting PaCO_2_ increase.^27^ The impact of hypercapnia in critically ill patients remains controversial. Clinical evidence is variable: some studies suggest that hypercapnia may be safe or even beneficial,^28–30^ whereas others have reported harmful effects.^31–35^ A recent meta-analysis reported that when permissive hypercapnia was employed to facilitate LPV, mortality was reduced, whereas hypercapnia occurring in patients already receiving LPV was associated with increased mortality compared with normocapnia.^31^ A secondary analysis of the LUNG SAFE study and a retrospective analysis of 3153 critically ill patients reported that hypercapnia is common among invasively ventilated patients and is not associated with mortality, concluding that there is no evidence of either harm or benefit from hypercapnia.^29 30^ Interpreting these findings remains challenging, and a prospective RCT is required to clarify the clinical implications of hypercapnia in critically ill patients.

This study has limitations. Blinding of clinicians and participants is not feasible due to the nature of the intervention. However, the protocol rigorously prespecifies how to compute and deliver the target RR, as well as safety thresholds for AEs, minimising performance and detection bias. Moreover, ventilator and patient management are carried out by clinicians and nurses whose primary objective is high-quality care and who have no interest in the trial outcomes. The study is conducted in a limited number of centres, which may restrict generalisability to other settings, healthcare teams, and patient populations. For instance, logistical challenges or rare AEs that could emerge in a large-scale RCT may not be observed. However, the trial has been designed to closely mirror the conditions of a future study, and all efforts have been made to anticipate potential challenges.

In conclusion, the PERMISS pilot trial will be the first RCT investigating whether a permissive LPV strategy aiming at lower RR and lower MP is feasible and safe. This trial will inform a larger, adequately powered, multicentre RCT investigating the impact of the permissive LPV on clinically relevant and patient-centred outcomes.

## Data Availability

Data sharing not applicable as no datasets generated and/or analysed for this study.
